# Iron deficiency in healthy, term infants aged five months, in a pediatric outpatient clinic: a prospective study

**DOI:** 10.1186/s12887-023-04277-7

**Published:** 2024-01-23

**Authors:** Nur Aida Adnan, Emer Breen, Chin Aun Tan, Crystal C Wang, Muhammad Yazid Jalaludin, Lucy Chai See Lum

**Affiliations:** 1 Pediatric Department, Hospital Tunku Azizah, Kuala Lumpur, Malaysia; 2 Department of Pediatrics, Faculty of Medicine, University Malaya, Kuala Lumpur, Malaysia; 3 Clinical Investigation Center, University of Malaya Medical Center, 5th Floor East Tower, Kuala Lumpur, Malaysia; 4 Occupational Safety and Health Unit, Hospital Tunku Azizah, Kuala Lumpur, Malaysia; 5 Weill Cornell Medicine, New York, NY USA

**Keywords:** Anemia iron deficiency, Breastfeeding, Ferritin, Prospective study

## Abstract

**Background:**

Iron deficiency (ID) is prevalent in Malaysian children. The incidence of ID in infants under 6 months of age is unknown. Our aim was to determine the prevalence of iron deficiency (ID) and iron deficiency anemia (IDA) in healthy, term infants aged below 6 months in our hospital population.

**Methods:**

A prospective longitudinal pilot study of mother-infant pairs was conducted on infants receiving routine immunizations in a mother and child clinic at a university hospital, in Kuala Lumpur, Malaysia. Mothers completed standardized questionnaires at 3- and 5-month postnatal visits. Maternal and infant full blood count, ferritin, and C-reactive protein (CRP) levels were measured at 3 months and for the infants repeated at 5 months. Infant anthropometric measurements were obtained at both visits. We conducted a univariate analysis to identify factors associated with ID and IDA.

**Results:**

Altogether, 91 mother-infant pairs were enrolled, with 88 completing the study. No infant had ID or IDA at 3 months; the lowest ferritin level was 16.6 µg/L. At 5 months, 5.9% (5/85) of infants had ID, and 2.4% (2/85) had IDA. Median (interquartile range) infant ferritin levels significantly declined from 113.4 (65.0–183.6) µg/L at 3 months to 50.9 (29.2–70.4) µg/L at 5 months, p < 0.001. Exclusive breastfeeding until 3 or 5 months was significantly associated with ID at 5 months (p = 0.020, and p = 0.008, respectively) on univariate analysis. The drop in ferritin between 3–5 months was significantly associated with weight and length gains between 0–3 months (p = 0.018, p = 0.009, respectively). Altogether, 14.3% of infants exclusively breastfed until 5 months developed ID. At 5 months, 3.4% of infants were underweight, 1.1% stunted, and 10.2% wasted.

**Conclusions:**

In exclusively breastfed term infants, ID occurred by 5 months. Early introduction of iron-rich foods should be considered in exclusively breastfed babies. A high prevalence of wasting suggests a calorie deficit in this population and will lead to stunting if not addressed.

## Introduction

In 2019, the global prevalence of anemia was estimated to be 40% in children aged 6 months to 5 years and 35% in East and Southeast Asia [1]. Iron deficiency anemia (IDA) is the most common anemia of childhood and contributes to approximately one-quarter to one-half of the anemia burden [1]. The prevalence of iron deficiency (ID) and IDA in those under six months is not well understood.

Iron plays a key role in oxygen transport, DNA replication, nerve myelination, neurotransmitter formation, and brain cell differentiation [2, 3]. In the first two years of life, persistent ID leads to impaired cognitive, motor, and social development, with detrimental effects on behavior, learning, and school performance [2–5].

The iron content of breast milk is low, and healthy, term, breastfed babies rely on their intrauterine iron endowment, which lasts until 4–6 months of age [5]. In healthy babies, declining ferritin levels postnatally have been associated with length and weight gains [6]. Maternal chronic disease (e.g., hypertension, diabetes), smoking in pregnancy, maternal ID during pregnancy, infant prematurity, or being small for gestational age increase the risk of infant ID [7].

Despite the harm that ID causes, there is insufficient evidence to suggest that universal supplementation of young children or pregnant women improves outcomes [5]. A 2015 Cochrane review highlighted that in populations with high anemia prevalence, iron supplementation during pregnancy reduces maternal anemia and ID at term and postnatally; however, the evidence only supports a borderline decreased risk of low-birth-weight babies and premature deliveries [8]. One study [9] in this review found that iron supplementation in pregnant Nigerian women significantly increased infant ferritin by 11 ug/L at six months postnatally.

There is some evidence to suggest that iron treatment improves cognitive function in anemic school-aged children, but this is less well established in those aged below 2 years with anemia or ID [5]. Indeed, iron supplementation has been associated with alteration to the gut microbiome, diarrhea, and particularly in children with normal iron levels, delayed growth, increased infection, and a potential decline in cognitive development [10, 11].

In 2019, for Malaysian children, the WHO estimated the prevalence of anemia to be 24.6% (9.7–40.1%) in those aged 6–59 months, with dietary ID responsible for 3.23% of total disability-adjusted life years and 14.85% of years of life lost due to disability [12]. A secondary analysis of the 2013 Nutrition Survey of Malaysian children (SEANUTS Malaysia) found the prevalence of anemia and ID to be 4% and 5.2%, respectively in primary school children [13]. However, these findings differ from those of smaller local studies where the prevalence of anemia and IDA was found to be 31.4% and 13.8%, respectively, among 261 rural school children aged 8–10 years in Sabah, East Malaysia [14], and 12.8% and 7.7%, respectively, in 249 school children aged 7–9 years in Kelantan, Peninsular Malaysia [15].

Studies in younger Malaysian children report higher prevalence of IDA at 27–39%; however, these studies are more than 10 years old, and one included only 20 children [16, 17]. Some of the Malaysian studies focused on rural [14, 16], predominantly low-income groups [14–17], with a high incidence of coexisting helminth infections [14, 16], poor sanitation, and lack of piped water [14]. Multivariate analysis showed significant associations between IDA and maternal education [16], low birth weight, and increased household size [17].

To date, no known study on ID and IDA in infants below the age of 6 months has been conducted in Malaysia. The primary objective of this study was to determine the prevalence of ID and IDA in a cohort of healthy, term infants aged 3–5 months in our university hospital in the capital city, Kuala Lumpur, and to identify factors associated with ID. The secondary objectives were to examine the growth of these infants and to check for any associations between ID and growth rates.

## Methods

### Study population, setting, and design

This was a prospective pilot study conducted in the Child Health Clinic at University Malaya Medical Centre, a tertiary university hospital in Kuala Lumpur, Malaysia, between 26 June, 2019 and 26 February, 2020. Altogether, 91 mother-infant pairs, who attended for routine growth and vaccination visits were invited to participate.

### Inclusion and exclusion criteria

Included were singleton infants delivered at term ($\ge$37 weeks’ gestation) in our hospital with birth weight ≥ 2.5 kg, who attended the Child Health Clinic, and were well during review. Infants who had any prior intensive care unit admissions, serious congenital malformations or illnesses, history of blood or exchange transfusions, or known family history of hematological disorders, such as thalassemia, were excluded from the study.

### Data collection

All medical-related information such as birth details, the mother’s medical history, and hemoglobin level at delivery were obtained from the hospital electronic database. At the first (3-month) and second (5-month) postnatal visits, a written questionnaire was completed by the parent, under the supervision of a trained nurse researcher. Data collected included combined household income, educational status, intake of maternal iron supplements in pregnancy and postnatally, and infant feeding practices.

Infant anthropometric measurements were recorded at both visits by a trained nurse, assisted by the research nurse. The infant’s weight was measured without clothes and diapers to the nearest 0.01 kg using a calibrated, digital weighing scale, Seca 334 (Seca Deutschland, Hamburg, Deutschland). The infant’s length was measured without clothes using a calibrated Harpenden Infantometer (Holtain Ltd, Wales, UK) to the nearest 0.1 cm. The head circumference was measured with a non-elastic plastic measuring tape to the nearest 0.1 cm.

The mothers had venous blood sampled at the 3-month visit, and the infants at the 3- and 5-month visits. Tests included a full blood count (FBC) analyzed using Sysmex XN-20 (Sysmex Corp., Kobe, Japan), C-reactive protein (CRP) using Siemens ADVIA 2400 Chemistry System (Siemens Healthineers, Siemens Healthcare GmBH, Erlangen, Germany), and ferritin using Siemens Centaur XP Immunoassay System (Siemens Healthineers, Siemens Healthcare GmBH, Erlangen, Germany).

### Sample size calculation

Based on past clinic attendance, a total of 100 babies were expected over the 25-week recruitment period. Since no local data were available for this age group, the estimated ID prevalence was taken to be 28.6%, as reported in a study of 5-month-old Peruvian infants [18]. Using the online Epitools Epidemiological Calculators [19] and setting the precision at 5% and confidence interval at 95%, the minimum sample size required was 77. Considering an anticipated dropout rate of 15%, 91 mother-infant pairs were required.

### Statistical analysis

Z-scores were calculated from the anthropometric measurements using the Ped (z) – Pediatric Calculator and the CDC/WHO reference data corrected for gestational age and sex [20]. Infants were classified as underweight if their weight for age was < -2 standard deviations (SD), stunted if their length-for-age was < -2 SD, wasted if their weight-for-length was < -2 SD, and severely wasted if their weight-for-length was < -3 SD from the median of the WHO reference population [21].

Using the WHO classification [22], ID among infants (0–23 months) and adults was defined as ferritin < 12 µg/L and < 15 µg/L, respectively if CRP $\le$5 mg/L, and ferritin <30 µg/L and <70 µg/L, respectively if CRP >5 mg/L. As per WHO definitions [23], anemia was defined as a hemoglobin <110 g/L for children aged < 5 years and pregnant women and <120 g/L for non-pregnant adults. IDA was said to occur when ID and anemia were present simultaneously.

Categorical data are described using numbers and percentages. The Kolmogorov–Smirnov test was used to assess for normal distribution. Normally distributed continuous data are described using mean and standard deviation (SD); if not normally distributed, median, and interquartile range (IQR) are used. If a serially measured variable fluctuated in its normality over time, the median and IQR were used for comparisons across all time points. Correlations between continuous data were checked using Spearman’s Rank correlation coefficient. The Mann-—Whitney U test was used to assess for significant difference between continuous variables in two independent groups and the Kruskal—Wallis test for more than two independent groups, with the Bonferroni posthoc test for pairwise comparisons. The Wilcoxon signed-rank test was used to assess significant differences between related groups. Univariate associations between the presence of ID at 5 months were explored, using the chi-squared test (Fisher’s exact test if n < 5) for categorical data. Data were analyzed using SPSS Statistics for Windows, version 23.0 (IBM Corp., Armonk, NY, USA). P-values < 0.05 were considered statistically significant.

## Results

Altogether, 91 infant-mother pairs were enrolled. These were reviewed when the infants were aged 3 months (Visit 1). Of these, 88 infant-mother pairs returned when infants were aged 5 months (Visit 2). Three pairs dropped out, because the mothers had decided to continue routine follow-up visits at a government-run child-health clinic.

### Maternal results

Maternal demographics, blood results, and iron status are shown in Table 1. The mean (SD) age of the mothers was 32.0 (5.0) years, and the sample fairly represented the distribution of Malaysia’s three main ethnicities. Household income was between Malaysian Ringgit (RM) 3000–6500 for 48.4% and represented typical income distribution at this time. Altogether, 96.7% (88/91) of the mothers reported taking antenatal iron supplements during pregnancy. In total, 28.6% (26/91) of mothers had antenatal complications, of which 84.6% had diabetes, hypertension, or both. In total, 22.5% (20/91) of mothers were anemic at delivery. At the 3-month visit, 12.1% (11/91) of mothers were anemic, 29.7% (27/91) had ID, and 4.4% (4/91) had IDA. At 3 months, 24.2% (22/91) of mothers were using iron supplementation, but this declined to 13.6% (12/88) at five months. Of the mothers with ID at 3 months, 22.2% (6/27) were on iron supplementation, versus 7.4% (2/27) at 5 months.

**Table 1:** Maternal demographics, blood results, and iron status

	Value
**Age**, year	
Mean (SD)	32.0 (5.0)
**Ethnicity**, n (%)	
Malay	53 (58.2)
Chinese	24 (26.4)
Indian	8 (8.8)
Others	6 (6.6)
**Education**, n (%)	
Secondary	20 (22.0)
Post-secondary	71 (78.0)
**Occupation**, n (%)	
Not employed	17 (18.7)
Employed	74 (81.3)
**Household income**, n (%)	
<RM 3,000	27 (29.7)
RM 3,000–6,500	44 (48.4)
RM > 6,500	20 (22.0)
**Antenatal complications**, n (%)	
None	65 (71.4)
Yes	26 (28.6)
**Antenatal iron supplements**, n (%)	
Yes	88 (96.7)
No	3 (3.3)
**Iron supplementation**, n (%)	
Visit 1	22 (24.2)
Visit 2	12 (13.6)
**Anemia/ID /IDA**, n (%)	
Anemia at delivery	20 (22.5)^1^
**Visit 1**	
Anemia	11 (12.1)
ID	27 (29.7)
IDA	4 (4.4)
ID and taking oral iron	6 (22.2)
**Visit 2**	
ID at Visit 1 and taking iron	2 (7.4)
**Blood test results**	
**Delivery**	
Hb (g/L), mean (SD)	116.2 (12.2)
**Visit 1**	
Hb (g/L), mean (SD)	129.5 (9.3)
Ferritin (µg/L), median (IQR)	53.3 (27.5–97.7)
CRP (mg/L) median (IQR)	2.17 (0.44–5.86)

Abbreviations: SD, standard deviation; RM, Ringgit Malaysia; ID, iron deficiency; IDA, iron deficiency anemia; Hb, hemoglobin; CRP, C-reactive protein

^1^2 cases missing data

## Infant results

Table 2 summarizes infant demographics, anthropometrics, feeding patterns, and blood results. There were 50.5% (46/91) male and 49.5% (45/91) female infants. The birth route was vaginal for 64.8% (59/91) and Caesarean section for 35.2% (32/91). At 3 and 5 months, 46.2% (42/91) and 39.8% (35/88) of infants, respectively, were exclusively breastfed. Of the 88 infants reviewed at 5 months, only one had been started on a complementary diet. This infant was exclusively formula fed at 3 and 5 months.

**Table 2:** Infant demographics, anthropometrics, feeding patterns and blood test results at 3 and 5 months

**Characteristic**	Age		
	Birth	3 months	5 months
Gender, n (%)			
Male	46 (50.5)		
Female	45 (49.5)		
**Gestation (weeks), n (%)**			
37–38	34 (37.4)		
38–39	22 (24.2)		
39–40	27 (29.7)		
>40	8 (8.8)		
**Birth route, n (%)**			
Caesarean section	32 (35.2)		
Vaginal delivery	59 (64.8)		
**Anthropometry**			
Weight, median (IQR)	3.1 (2.8–3.4)	5.9 (5.5–6.5)	6.9 (6.4–7.6)
Length, median (IQR)	48.0 (46.5–49.0)	61.0 (58.7–62.6)	65.0 (63.5–67.0)
Head circumference, median (IQR)	33.0 (32.0–34.0)	40.0 (39.5–41.0)	42.0 (41.0–43.0)
z-score weight-for-age, median (IQR)	-0.31 (-1.06–0.17)	-0.27 (-0.75–0.34)	-0.33 (-1.1–0.26)
z-score length-for-age, median (IQR)	-0.93 (-1.52–-0.08)	0.11 (-0.52–1.20)	0.20 (-0.49–1.10)
z-score HC for age, median (IQR)	-0.76 (-1.59–-0.2)	0.24 (-0.43–0.84)	0.07 (-0.38–0.76)
z-score weight-for-length, median (IQR)	0.44 (-0.11–0.95)	-0.25 (-1.11–0.32)	-0.52 (-1.43–0.37)
Underweight (weight-for-age <-2 SD), n (%)	0/91 (0)	1/91 (1.1)	3/88 (3.4)
Stunting (length-for-age <-2 SD), n (%)	13/91 (14.3)	2/91 (2.2)	1/88 (1.1)
Wasting (weight-for-length <-2 SD), n (%)	0/84 (0)	8/91 (8.8)	9/88 (10.2)
Severe wasting (weight-for-length <-3 SD), n (%)	0/84 (0)	3/91 (3.3)	4/88 (4.5)
**Feeding pattern**			
Exclusively breast feeding		42/91 (46.2)	35 (39.8)
Mixed feeding		38/91 (41.8)	27 (30.7)
Fully formula feeding		11/91 (12.1)	26 (29.5)
Supplementary feeding		0/91 (0)	1 (1.1)
**Blood test results**			
Hb (g/l) mean (SD)		113.4 (8.1)	118.9 (8.5)
Ferritin (µg/l), median (IQR)		113.4 (65.0–183.6)	50.9 (29.2–70.4)
CRP (mg/l), median (IQR)		0.03 (0.00–0.12)	0.04 (0.00–0.24)
**Anemia/ID /IDA**			
Anemia		27/90 (30.0)	14/86 (16.3)
ID		0/90 (0)	5/85 (5.9)
IDA		0/90 (0)	2/85 (2.4)

Abbreviations: SD, standard deviation; IQR, interquartile range; HC, head circumference; Hb, hemoglobin; CRP, C-reactive protein; ID iron deficiency; IDA, iron deficiency anemia

ID was defined as ferritin < 12 µg/L if CRP ≤ 5 mg/L and ferritin < 30 µg/L if CRP > 5 mg/L. Anemia was defined as a hemoglobin < 110 g/L. IDA was said to occur when ID and anemia were present simultaneously

The median birth z-scores for weight, length, and head circumference were all under the 50th centile (Table 2). At 3 months the average infant grew along a higher centile than the birth centile, with the median length and head circumference surpassing the 50th centile. By 5 months, the head circumference and length growth rate maintained on a similar trajectory, with the median weight remaining below the 50th centile. The median z-score weight-for-length of the infants fell significantly from above the 50th centile at birth to below the 50th centile at 3 months (p < 0.001) and fell further at 5 months, (p > 0.05).

For gender differences and growth see Table 3. At birth there were no significant differences in anthropometric measurements between male and female infants. At 3 and 5 months, male infants had significantly greater median weight and length compared with female infants; for head circumference, this increase was significant at 3 months only and borderline at 5 months (p = 0.061). There were no significant gender differences in change in median z-score from birth to 3 months, birth to 5 months, or between 3 and 5 months (all p > 0.05).

**Table 3:** Gender differences in anthropometry, hemoglobin, and ferritin levels of infants aged 3 and 5 months

	Birth			3 months			5 months		
	Male`	Female	p value	Male	Female	p value	Male	Female	p value
	n = 46	n = 45		n = 46	n = 45		n = 43	n = 45	
Weight (kg), median (IQR)	3.1 (2.8–3.4)	3.1 (2.8–3.3)	0.621	6.0 (5.7–6.5)	5.6 (5.2–6.2)	**0.001**	7.0 (6.6–7.6)	6.6 (6.1–7.3)	**0.003**
Body length (cm), median (IQR)	48.0 (46.9–50.0)	48.0 (46.5–49.0)	0.623	61.9 (60.1–63.3)	59.3 (57.9–61.7)	**< 0.001**	65.8 (64.3–68.6)	64.5 (62.5–66.2)	**0.001**
HC (cm), median (IQR)	33.0 (32.0–34.5)	33.0 (32.0–33.0)	0.051	40.5 (40.0–41.1)	39.8 (39.0–41.0)	**0.007**	42.5 (41.5–43.0)	41.8 (41.0–42.8)	0.061
				n = 46	n = 44		n = 42	n = 44	
Hb (g/L), mean (SD)				112.8 (7.9)	114.0 (8.5)	0.523	117.3 (7.4)	120.4 (9.3)	0.082
				n = 46	n = 45		n = 41	n = 44	
Ferritin (µg/L), median (IQR)				88.9 (59.4–149.6)	145.3 (76.1–240.3)	**0.039**	40.1 (25.9–60.8)	57.2 (31.5–82.4)	0.095

Abbreviations: IQR, interquartile range; SD, standard deviation; HC, Head circumference; Hb, hemoglobin

At birth, 14.3% (13/91) of infants were stunted (see Table 2). At 3 months 1.1% of infants were underweight, 2.2% stunted, 8.8% wasted, and 3.3% severely wasted. At 5 months 3.4% of infants were underweight, 1.1% were stunted, 10.2% wasted, and 4.5% severely wasted. In all these categories the percentage of infants within each group increased with time, apart from the stunted category where it fell from birth–3 months and from 3–5 months. There were no significant gender differences or differences in feeding patterns between underweight, stunted, and wasted infants.

Infant blood test results are included in Table 2. Due to insufficient samples or samples clotting, at 3 months (n = 91), there was no hemoglobin level available for one infant, who did have a ferritin level recorded, while at 5 months (n = 88, due to three mother-infant pairs that dropped out of the study), two infants had no blood results recorded, while one infant had a hemoglobin level but no ferritin level available; therefore, only 86 and 85 infants were assessed for anemia and ID, respectively, at 5 months. Altogether, 30% (27/90), and 16.3% (14/86) of infants were anemic at 3 and 5 months, respectively. Maternal hemoglobin at delivery was weakly but significantly correlated with infant hemoglobin at 3 and 5 months (r = 0.276, p = 0.009, and r = 0.269, p = 0.013, respectively). At 3 months, an anemic mother was significantly more likely to have an anemic infant (p < 0.001), and maternal hemoglobin levels were significantly correlated with infant hemoglobin levels (r = 0.358, p = 0.001). There was no significant relationship between maternal ferritin levels at 3 months and infant ferritin levels at 3 or 5 months. No infant had ID or IDA at 3 months, although the lowest ferritin level was 16.6 µg/L; the highest ferritin level at 3 months was 909.6 µg/L, demonstrating a wide variation in ferritin levels. At 5 months, 5.9% (5/85) of infants had ID; two of these infants also had anemia i.e., 2.4% of all infants (2/85) had IDA. Infant ferritin levels at age 3 months were significantly strongly correlated with infant ferritin levels at age 5 months (r = 0.734, p < 0.001).

Table 3 also compares ferritin levels between male and female infants. Male infants had lower median ferritin at 3 and 5 months compared with females, although this difference was only significant at 3 months; median ferritin was 88.9 (59.4–149.6) µg/L in males and 145.3 (76.1–240.3) µg/L in females (p = 0.039) at 3 months, and 40.1 (25.9–60.8) ug/L in males and 57.2 (31.5–82.4) in females (p = 0.095) at 5 months.

**Fig. 1:**
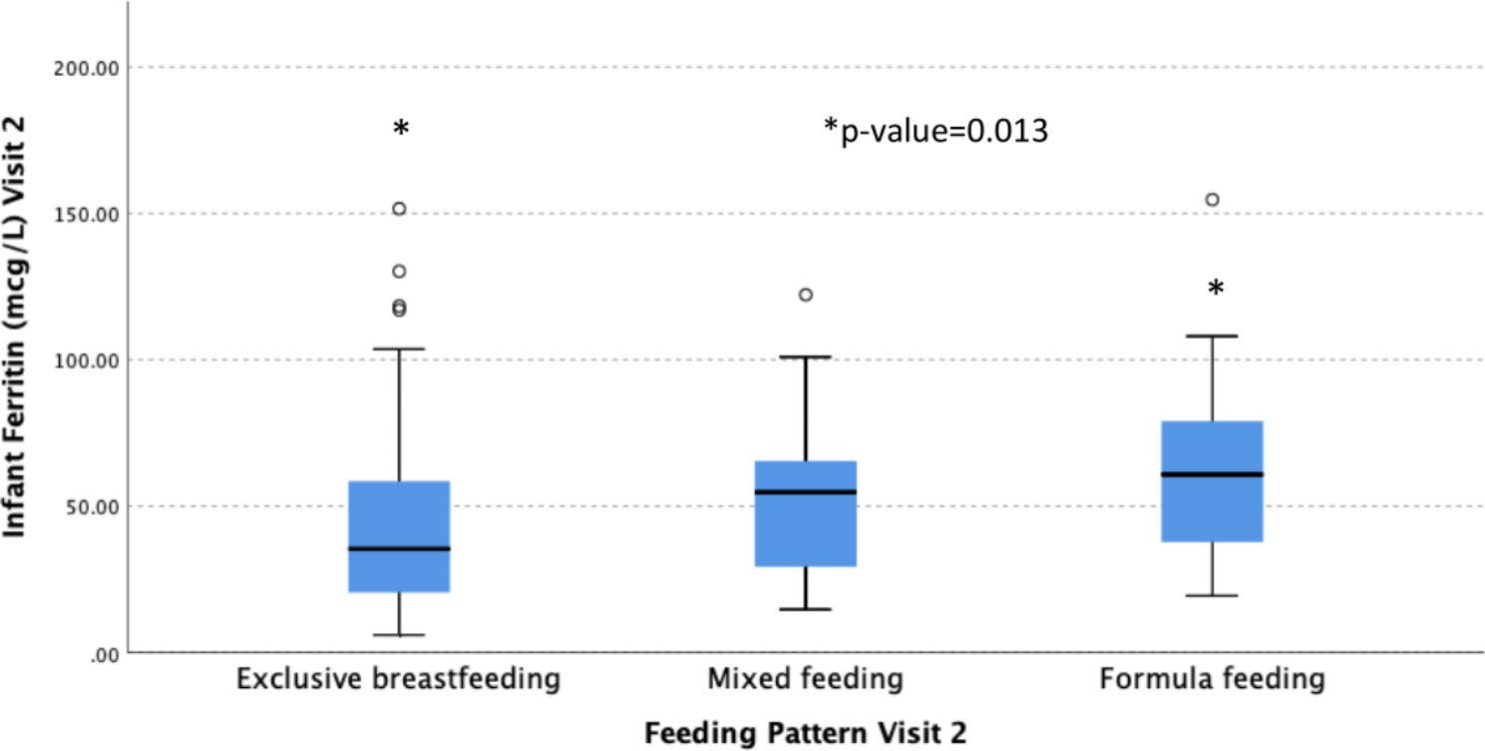
Comparisons of ferritin levels between feeding groups at 5 months *Kruskal–Wallis test and Bonferroni posthoc test showed a significant difference in ferritin levels between exclusively breastfed and formula-fed groups (p = 0.013)

A comparison of hemoglobin and ferritin levels between groups of infants with different feeding patterns at 3 and 5 months is shown in Table 4. There were no statistically significant differences in hemoglobin levels between exclusively breastfed, mixed-fed, and exclusively formula-fed groups at 3 or 5 months. Hemoglobin levels rose across all feeding groups from 3 to 5 months, while ferritin levels fell. The lowest median ferritin levels were in the exclusively breastfed group and highest in the exclusively formula-fed group, with the mixed-feeding group in between; this between-group difference was not significant at 3 months (p = 0.090) but was at 5 months (p = 0.016). There was a statistically significant difference between median ferritin levels for the exclusive breast- and formula-fed infants at 5 months (p = 0.013) but not between exclusive breast- and mixed-fed infants (p = 0.364) or between formula- and mixed-fed infants (0.635). See Fig. 1. Median (IQR) infant ferritin levels in exclusively breastfed infants declined from 103.1 (50.9–116.9) µg/L at 3 months to 35.4 (19.9–60.6) µg/L at 5 months at a rate of 1.13 µg/L/day or 1.1%/day.

**Table 4:** Infant hemoglobin and ferritin levels according to feeding pattern at ages 3 and 5 months

	3 months			5 months		
	**n/total (%)**	**Hb (g/L), mean (SD)**	**Ferritin (µg/L), median (IQR)**	**n/total (%)**	**Hb (g/L), mean (SD)**	**Ferritin (µg/L), median (IQR)**
Exclusively breast feeding	42/91 (46.2)	115.1 (7.8)	103.1 (50.9–116.9)	35/88 (39.8)	118.6 (8.2)	*35.4 (19.9–60.6)
Mixed feeding	38/91 (41.8)	110.7 (8.3)	125.8 (76.1–171.4)	27/88 (30.7)	118.8 (7.6)	54.7 (29.2–67.0)
Exclusively formula feeding	11/91 (12.1)	116.2 (6.8)	183.6 (91.2–241.3)	26/88 (29.5)	119.3 (9.8)	*60.8 (37.6–83.6)
p-value for difference among feeding groups		0.069	0.090		0.958	**0.016**

Abbreviations: Hb, hemoglobin; SD, standard deviation; IQR, interquartile range

*Bonferroni posthoc test shows a statistically significant difference between exclusively breast-feeding and exclusively formula-feeding groups (p = 0.013)

There were weak but significant associations between ferritin drop between 3 and 5 months and weight and length gain from 0 to 3 months (r=-0.257, p = 0.018 and r=-0.280, p = 0.009, respectively). The association between ferritin drop between 3–5 months and head circumference gain 0–3 months was borderline (p = 0.069) There was no significant associations between change in ferritin and growth parameters between 3–5 months.

For the six infants with no 5-month ferritin levels recorded, there was no significant difference between the median ferritin levels at the 3-month visit compared with the infants who did have a second ferritin measured (p = 0.644). All three who did not return for the second visit received mixed breast and formula milk at 3 months. Of the three who returned for the second visit and did not have ferritin levels measured, two were mixed fed and only one was exclusively breastfed.

### Univariate analysis

A comparison of variables between ID and non-ID infant groups is shown in Table 5. There were no significant differences between ID and non-ID infants in the period of gestation, birth weight, mode of delivery, and gender, nor in maternal factors such as age, antenatal complications, ethnicity, income group, employment status, consumption of iron supplements during the pregnancy, presence of anemia at delivery, and presence of ID, or IDA. Exclusive breastfeeding at 3 and 5 months (p = 0.02, and p = 0.008, respectively) and mean hemoglobin at 5 months (111.8 g/dL vs. 119.4 g/dL, p = 0.023) were significantly associated with ID at 5 months on univariate analysis.

**Table 5:** Comparison of variables between infants with and without iron-deficiency aged 5 months

Variable	Iron deficient* infants	Non-iron deficient infants	p-value
**Gestational age**			
37 to < 39 weeks	60% (3/5)	60% (48/80)	1.0
≥ 39 weeks	40% (2/5)	40% (32/80)	1.0
**Caesarean section**	60% (3/5)	30% (24/80)	0.321
**Pregnancy complications present**	40% (2/5)	27.5% (22/80)	0.618
**Mother Malaysian Chinese**	60% (3/5)	22.5% (18/80)	0.587
**Family income group > RM 6500**	40% (2/5)	21.3% (17/80)	0.583
**Mother post-secondary education**	100% (5/5)	77.5% (62/80)	0.356
**Mother employed**	100% (5/5)	80% (64/80)	0.578
**Pregnancy iron**	100% (5/5)	96.3% (77/80)	1.0
**Mother iron supplementation at**:			
3 months	60% (3/5)	21.3% (21/80)	0.082
5 months	0% (0/5)	15% (12/80)	1.0
**Mother anemia/ID/IDA**			
Anemia at delivery	20% (1/5)	23.1% (18/80)	1.0
Anemia at 3 months	0% (0/5)	11.3% (9/80)	0.563
ID at 3 months	20% (1/5)	30% (24/80)	1.0
IDA at 3 months	0% (0/5)	5% (4/80)	1.0
**Male gender**	60% (3/5)	47.5% (38/80)	0.669
**Exclusive breast feeding at**:			
3 months	100% (5/5)	43.8% (35/80)	**0.020**
5 months	100% (5/5)	36.3% (29/80)	**0.008**
**Maternal age (years), mean (SD)**	34.2 (3.4)	31.8 (5.1)	0.203
**Infant weight (kg), median (IQR) at**:			
Birth	3.1 (2.8–3.3)	3.1 (2.8–3.3)	0.580
3 months	6.4 (5.5–6.6)	5.8 (5.5–6.3)	0.088
5 months	7.2 (7.2–7.6)	6.8 (6.4–7.5)	0.520
**Infant length (cm), median (IQR) at**:			
Birth	48.0 (45.9–49.0)	48.0 (46.5–49.0)	0.486
3 months	64.0 (59.5–64.5)	61.0 (58.7–62.4	0.209
5 months	65.7 (64.5–67.4)	65.0 (63.5–66.9)	0.425
**HC (cm), median (IQR) at**:			
Birth	32.0 (31.8–33.3)	33.0 (32.0–34.0)	0.183
3 months	40.5 (39.8–41.5)	40.0 (39.5–41.0)	0.431
5 months	42.0 (41.5–43.5)	42.0 (41.0–43.0)	0.841
**Change z-score, median (IQR)** **Weight**:			
0–3 months,	0.51 (-0.07–0.97)	0.05 (-0.49–0.72)	0.309
0–5 months	0.14 (-0.16–0.85)	-0.17(-0.77–0.83)	0.360
0–5 months	-0.13 (-0.28–-0.06)	-0.10 (-0.42–0.16)	0.647
**Length**:			
0–3 months,	1.95 (1.33–2.15)	1.15 (0.16–2.07)	0.179
0–5 months	1.50 (0.33–2.72)	1.08 (0.15–2.14)	0.513
3–5 months	0.17 (-1.65–0.90)	0.14 (-0.92–0.85)	0.575
**HC**:			
0–3 months,	2.33 (0.53–2.99)	0.87 (0.15–1.72)	0.130
0–5 months	2.49 (0.32–2.88)	0.98 (0.16–1.71)	0.151
3–5 months	-0.02 (-0.46–0.24)	-0.06 (-0.74–0.46	0.977
**Weight/length**:			
0–3 months,	-1.33 (-1.48–-0.83).	-0.95 (-1.82–-0.75)	0.863
0–5 months	-0.98 (-2.27–0.46)	-1.13 (-1.96–-0.12)	0.602
3–5 months	-0.64 (-1.20–-0.12)	0.01 (-1.13–0.50)	0.859
**Infant Hb (g/dL), mean (SD) at**:			
3 months	114.8 (5.3)	113.8 (8.2)	0.855
5 months	111.8 (5.1)	119.4 (8.5)	**0.029**

Abbreviations: RM, Ringgit Malaysia; ID, iron deficiency; IDA, iron-deficient anemia; SD, standard deviation; IQR, interquartile range; HC, head circumference; Hb, hemoglobin

*Iron deficiency among mothers was defined as ferritin < 15 µg/L if CRP ≤ 5 mg/L, and ferritin < 70 µg/L if CRP > 5 mg/L. Anemia was defined as a hemoglobin < 110 g/L for in pregnant women and < 120 g/L for non-pregnant adults. IDA was said to occur when ID and anemia were present simultaneously

At birth, the median weight and length were the same for both ID and non-ID groups; the head circumference was lower in the ID group compared with the non-ID group, though this difference was not significant (p > 0.05). At 3- and 5-months median weight and length were higher in the ID group than the non-ID group, though this was not significant, while the head circumference achieved parity with the non-ID group at 5 months. There were no significant associations between changes in median z-scores for weight, length, or head circumference at birth to three, birth to five, or three to five months between ID and non-ID groups. Calculating multivariate models was impossible due to the limited data for ID babies (n = 5) and there was a 100% correlation of ID with breastfeeding at 5 months. Of all babies exclusively breastfed until 5 months, 14.3% (5/35) had ID and 5.7% (2/35) had IDA. One infant with ID at 5 months was stunted at birth and another was wasted at 5 months, but there were no significant associations between being underweight, stunted, wasted, or none of these and the presence of ID at 5 months.

## Discussion

This is the first-known work detailing ferritin levels in healthy term, Malaysian infants aged below six months. We showed that for infants who attended this Child Health Clinic in Kuala Lumpur, Malaysia, the incidence of ID, and IDA was 5.9% and 2.4%, respectively, at age 5 months. Exclusive breastfeeding at 3 and 5 months was significantly associated with ID at 5 months, with 14.3% of exclusively breastfed infants developing ID at that time point.

Our finding that ID develops before the age of 6 months is consistent with other studies. Indeed, in prospective studies, including those in high-income settings, the IDA prevalence increases beyond six months, because introducing solids cannot replenish stores quickly enough [6, 24]. In a midwestern US-based prospective study of healthy, breastfed infants whose ferritin levels were tracked from 1 month of age, there was a similar ID prevalence ID, 5.3% at 5.5 months, with a four-fold rise in ID prevalence by 12 months [6]. A similar prevalence of ID, and four-fold rise by one year, was seen in a cross-sectional study of Northern Taiwanese infants [25]. All infants aged under 6 months who developed ID or IDA in this latter study were exclusively breastfed, as per our findings. While there is much individual country variation in the prevalence of ID and IDA in infancy, we can extrapolate based on the American and Taiwanese studies with similar findings of ID, that one-fifth of our infants could be ID by one year of age.

Infant ferritin levels varied widely at 3 months and were not significantly correlated with the maternal ferritin measured simultaneously. Previous studies have shown similar large variations in infant ferritin, with no evidence of a direct correlation between maternal pregnancy ferritin and infant iron stores except in severe cases [6]. Our infant ferritin levels significantly declined from 3 to 5 months as the intrauterine endowment was depleted. This too has been documented elsewhere [6, 7]. The 1.1% daily rate of decline we found between 3–5 months in exclusively breastfed infants was identical to that discovered between 2–5.5 months in the US study of similarly fed infants [6]. In addition, we showed a significant association between an infant’s ferritin at 3 months and 5 months, suggesting that if a ferritin level could be determined below which the risk of future ID is high, then it may be possible to predict and prevent the onset of ID. In the American study, researchers identified a 2-month ferritin level of 65 ug/L below which 42% developed ID (defined as ferritin < 10 µg/L) before 6 months [6]. In our study at 3 months, 25.2% (23/91) ferritin levels were less than 65 µg/L.

We showed that infant male ferritin levels were lower than female levels; this was significant at three months only, possibly due to small numbers. Male infants have been shown elsewhere to have lower ferritin than females of the same age, which is only partly explained by an increased growth rate [26]. We showed significantly greater weight, length, and head circumference for male infants compared to females but were unable to show significant gender differences in growth rates, possibly again because the study was not powerful enough.

Almost a third of our pregnant mothers had chronic disease complications (the majority were diabetes and hypertension). This is higher than the national prevalence in pregnant Malaysian women–19.3% for diabetes and hypertension combined [27]–and suggests selection bias due to the referral of high-risk pregnancies to our hospital. Although we were unable to find a significant association between chronic disease in pregnancy and reduced ferritin levels, this has been found in pregnancies complicated with diabetes and hypertension [7] and is thought to be due to a disruption of normal placental function and increased iron demand through stimulation of erythropoiesis by chronic hypoxia [28]. It is likely that with a more powerful study, these associations could be found.

We discovered significant univariate associations between maternal hemoglobin at delivery with infant hemoglobin at 3 and 5 months. Altogether, 20% of pregnant women were anemic at delivery, with 11.3% anemic at 3 months postpartum. A systematic review of studies examining the prevalence of anemia in pregnant Malaysian women showed wide variation in rates from 57% in rural Terengganu to 19.3% in Kuala Lumpur [29]. In Malaysia, it is routine practice for pregnant women to take 30 mg/day prophylactic, elemental iron supplementation from the first antenatal visit throughout pregnancy. This is available for free at government clinics; however, poor compliance was found in more than a third of Terengganu women and was associated with a significantly higher incidence of pregnancy anemia [30]. In Jordan, where the incidence of pregnancy anemia is high, infants of women with pregnancy anemia were significantly more likely to develop IDA themselves from 9 months, compared with infants of non-anemic pregnant women, even though their cord ferritin levels, and apparent iron endowment, were similar across both groups at birth [31].

One-third of mothers had ID at 3 months postpartum, while only 22.2% of these were on iron supplementation. While infant ferritin levels decline postnatally, maternal ferritin levels rise in the postpartum period [24]. The high incidence of maternal ID at 3 months is concerning and has implications, even if not associated with anemia, for the mother’s energy levels [32], ability to produce breast milk, bonding with her new-born, and for future pregnancies beginning in an-iron deficient state. Among non-anemic women with and without ID in early pregnancy, those with ID were significantly more likely to have ID in later pregnancy and postnatally despite iron supplementation and, even more concerningly, to have babies with significantly lower birthweight [33]. Although we did not show that ID was correlated with increased growth rate of infants, it is likely that our study was not powerful enough to detect this. We did, however, show that ferritin decline at 3–5 months was significantly associated with weight and length gain at 0–3 months. Other studies have shown associations between faster growth rates (in length, weight, and head circumference) and iron depletion [6, 34, 35].

Approximately, 1% of infants were stunted and 10% wasted at 5 months, with 5% being severely wasted, suggesting our infant population appears to be of an appropriate length but skinny. Wasting is not recorded on infant centile charts; thus, typically it is not measured in infants under 6 months. One review of 21 low- and middle-income countries estimated the prevalence of wasting to be 15% [36]. Since Malaysia is an upper-middle-income country, a 10% prevalence of wasting seems credible. Similar findings were observed in the Malaysian National Health and Morbidity Survey, 2019, in which the prevalence of wasting in children under 5 years was 9.7% [37]. If this wasting is not addressed adequately stunting will result. While the prevalence of stunting was low in our study, the prevalence of stunting in children aged under 5 years in Malaysia has reached 21.8% [37]. Failing to consider that wasting starts in early infancy is a public health opportunity missed and has implications for morbidity and mortality [38].

Evidence supports that breastfeeding is beneficial to the infant as it enhances sensory and cognitive growth, reduces the risk of obesity in later life, protects against infection and chronic disease, and reduces the maternal risk of reproductive cancers [39]. Malaysia follows the WHO’s advice in recommending exclusive breastfeeding until 6 months of age. Despite this WHO recommendation, in many high-income countries, most infants start complementary feeding earlier than six months due to the risk of ID. Indeed, the American Academy of Pediatrics recommends that at 4 months of age, full-term, exclusively breastfed infants commence iron supplementation until an iron-rich diet is provided [2].

In our study, all infants that developed ID at 5 months were exclusively breastfed, and there was a significant difference in ferritin levels between exclusively breastfed and formula-fed infants. While we could not exclude confounding factors, e.g., maternal ID, this makes sense given the fortification of formula milk with iron, and other studies showing a higher risk of ID and anemia in infancy with longer durations of exclusive breastfeeding [40, 41]. In a study of term, breastfed infants in Newfoundland, those receiving iron from birth until six months of age showed higher hemoglobin and mean corpuscular volume at six months and improved visual acuity and psychomotor development at 13 months compared with those not receiving supplemented iron [42]. This suggests that iron supplementation under six months may help prevent adverse effects in breastfed populations. In Thailand, introducing iron supplementation at 4 months to exclusively breastfed babies decreased the incidence of ID and IDA at six months [43].

Our results suggest, that in healthy term babies, exclusive breastfeeding until 6 months may not be the best recommendation to prevent ID. While iron supplementation of breastfed babies is one option, early weaning before six months with the inclusion of iron-rich, nutritionally dense foods should be considered in developmentally ready, breastfed Malaysian infants. Indeed, this is an opportunity for early introduction of potential allergens and a wider range of fruit and vegetables [44, 45].

### Strengths and limitations

The strengths of this study include that it was a prospective study, which followed infants at two time points aged below 6 months, and there was minimal loss to follow-up (n = 3). We chose 3 and 5 months to check for ID because the infants were returning at that time for routine vaccinations, thus helping limit loss to follow-up, and so that we could determine iron levels before the introduction of solid foods at 6 months. Limitations include that we had only a small number of participants, as the sample size calculation was based on a population with a much higher prevalence of ID than our ours; thus, we were unable to exclude confounding factors that could influence the relationship between ID and breastfeeding. Furthermore, we found that the prevalence of stunting decreased at 3 months (2.2%) compared with birth levels (14.3%). This may be explained by the fact that midwives measured length at birth prior to the study commencement; this may not be as accurate as those recorded by the trained research nurse. In addition, we did not assess the iron or smoking status of the mother in pregnancy, measure compliance with pregnancy iron supplementation, or consider previous pregnancies in our analysis, all of which can increase the risk of ID. However, according to the National Health and Morbidity Survey in 2015, only 1.4% of Malaysian women aged 15 and over smoked [46]. We plan to continue our research and recruit more mother-infant pairs to reduce the chances of confounding. An additional limitation is that our unique population profile (city-dwelling, middle-income group with a greater percentage of high-risk pregnancies) may affect the generalizability of results.

Further research is needed to determine whether screening for ID should be considered in exclusively breastfed babies at or before 5 months, as this would meet many of the Wilson and Jungner criteria [47]. Future studies should include more rural areas of Malaysia, where the incidence of maternal anemia is typically higher, so that a true understanding of the prevalence of ID in young infants across the whole country is ascertained.

## Conclusions

Iron deficiency occurred in 14.6% of exclusively breastfed healthy term infants by age 5 months. Additionally, 10% were wasted at 5 months. These have potential negative implications for an infant’s physical, developmental, cognitive growth, morbidity, and mortality and are important public health opportunities not to be ignored. While we were not able to exclude confounding, the introduction of iron-rich, nutrient-dense foods should be considered in exclusively breastfed infants from age 4 months.

## Data Availability

The datasets used and/or analyzed during the current study are available from the corresponding author on reasonable request.

## List of abbreviations

CRP: C-reactive protein
FBC: Full blood count
ID: Iron deficiency
IDA: Iron deficiency anemia
IQR: Interquartile range
RM: Ringgit Malaysia
SD: Standard deviation
SEANUTS: South East Asian Nutrition Survey
